# Application of electrocoagulation process for the disposal of COD, NH_3_-N and turbidity from the intermediate sanitary landfill leachate

**DOI:** 10.1007/s11356-024-31937-7

**Published:** 2024-01-13

**Authors:** Aysenur Ogedey, Ensar Oguz

**Affiliations:** 1 Civil Engineering Department, Munzur University, 62000 Tunceli, Turkey; 2 Environmental Engineering Department, Atatürk University, 25240 Erzurum, Turkey

**Keywords:** Intermediate leachate, Electrocoagulation, COD, NH_3_-N, Turbidity

## Abstract

This study aims to determine the COD, NH_3_-N and turbidity disposal efficiencies from leachate in the Bingöl landfill and highlight the electrocoagulation (EC) process’s performance in removing these pollutants. After establishing that landfill leachate was intermediate aged, its characteristics were identified using physical, chemical and elemental analyses. Six parallel-connected electrode plates with stainless steel as the cathode and aluminium as the anode were used to construct an electrocoagulation cell. After a 40-min treatment interval, the optimal disposal efficiencies for COD and turbidity from the leachate were determined to be 87% and 62%, respectively, at pH 5. Following a 40-min reaction, BOD_5_ concentration and BOD_5_/COD ratio were determined to be 85.75 mg O_2_/L and 0.64, respectively, at pH 5. At a NaCl concentration of 10 mM, the optimum disposal efficiency for NH_3_-N was determined to be 33%. The reaction kinetics matched pseudo-first-order (PFO) kinetics due to high correlation coefficients (*R*^2^ = 0.93–0.99) in removing COD, NH_3_-N and turbidity under different experimental conditions. The optimal reaction rate constants were determined as 2.93 × 10^−2^ min^−1^, 1.92 × 10^−2^ min^−1^ and 7.3 × 10^−3^ min^−1^ for the disposal of COD, NH_3_-N and turbidity, respectively. Energy consumption, unit energy consumption and total consumption cost rose in the EC process when the current density was augmented from 15 to 25 mA/cm^2^.

## Introduction

Due to population expansion, increased population density in urban areas and changing consumption practices, urban civilization produces an exponentially increasing amount of municipal solid waste (Fernandes et al. 2012). Landfills, which are used in many countries, are currently the standard practice for disposing of municipal solid waste. This standard practice results in the formation of leachate, a very complicated effluent. It comprises a range of heavy metals, as well as organic and inorganic materials, some of which are resistant and dangerous, and has a colour and smell (Öman and Junestedt 2008; Eggen et al. 2010). However, this process could cause certain environmental issues if the waste is not properly handled. As an illustration, when rainwater percolates into a landfill, it produces an effluent that is polluted with organic, inorganic and microbiological particles and may affect surface water and groundwater (Oumar et al. 2016). Environmental factors, including hydrology and atmospheric conditions, may influence the leachate’s quantity and quality. Additionally, the age of the landfill, the design of the covering and the method of operation can influence the characteristics of the generated leachate (Umar et al. 2010).

The age of the landfill affects the categorization of leachate: young leachate, found in landfills less than 5 years old; intermediate leachate, found in landfills between 5 and 10 years old; and old leachate, found in landfills older than 10 years (Bilgin et al. 2023). The ratio of BOD_5_ to COD (BOD_5_/COD) in young landfill leachate is 0.5–1.0, intermediate leachate is 0.1–0.5 and old leachate is less than 0.1. Since the BOD_5_/COD ratio is a direct indicator of the leachate’s degree of biodegradability among the other leachate properties, it is frequently cited as the best indicator of landfill leachate age (Verma and Kumar 2018). Leachate infiltrates and mixes with the soil, surface waterways and subsurface waters if it is not properly collected, stored and treated. This results in major environmental issues. Leachate requires to be regularly collected and disposed of; thus, it must undergo through the necessary treatment process before being released into the environment.

A number of treatment methods, including biological processes (Li et al. 2017), osmosis (Iskander et al. 2017), coagulation and flocculation processes (Wang et al. 2015), flotation methods (Adlan et al. 2011), adsorption (Hur and Kim 2000), membrane processes (Ahn et al. 2002), chemical precipitation (Erabee et al. 2018), chemical oxidation (Derco et al. 2010), advanced oxidation processes (Zhang et al. 2012; Chys et al. 2015), electro-Fenton (Zhang et al. 2014) and the Fenton process (Vallejo et al. 2013), have been used to treat landfill leachate. But, there are several drawbacks to these methods, such as transferring from one phase to another, high operational costs, diminishing process performance and ineffective pollutant removal. In order to effectively remove pollutants from landfill leachate, it is imperative to develop and implement a treatment procedure that is both economical and effective.

The electrocoagulation (EC) process has advantages such as ease of installation and automation, simplicity of equipment, short retention time and minimal sludge formation. Thus, it is regarded as an effective landfill leachate treatment technology since it may be utilised to treat a wide range of pollutants without the need for chemicals (Chen 2004; Ding et al. 2021). EC’s strong electrical conductivity and concentration of chloride make it a viable alternative for treating landfill leachate (Labanowski et al. 2010). The presence of chloride ions in the effluent regulates electrode dissolution by raising solution conductivity, which can reduce energy usage.

Some researchers have focused on treating landfill leachate using the EC process (Ihara et al. 2004), and several have utilised aluminium electrodes and iron electrodes for this purpose (Li et al. 2011; Pirsaheb et al. 2016; Rookesh et al. 2022). Due to their affordability and accessibility, iron and aluminium electrodes are frequently utilised in landfill leachate treatment. When compared to iron, the Al electrode demonstrated a greater elimination of COD, colour, turbidity and total nitrogen (Bouhezila et al. 2011). Using an Al electrode, previous investigations revealed that around 80–85% of colour, 87–95% turbidity, 40–66% COD and 84–94% BOD_5_ could be removed (Ricordel and Djelal 2014; Oumar et al. 2016; Pirsaheb et al. 2016; Galvão et al. 2020). However, in certain instances, it proved very challenging to remove the nitrogenous pollutants from the leachate in the EC process. According to a research, EC process with Al anode (Bouhezila et al. 2011) removed only 40% of nitrate while Fe anode (Li et al. 2011) removed 38.6% of ammonia nitrogen. EC process has proved effective in reducing suspended particles, colloids and high molecular weight organic compounds from wastewater, particularly landfill leachate (Dia et al. 2017).

The purpose of this research is to propose the electrocoagulation process as a treatment alternative for concurrently treating the pollution generated by COD, ammonia nitrogen (NH_3_-N) and turbidity, which are present in high concentrations in the intermediate leachate of the sanitary landfill in Bingöl. This investigation is notable because it employs a well-known purification process to treat intermediate leachate in a different location. The performed literature research revealed that there had been no studies on the use of the EC process for treating leachate arising from the Bingöl landfill. After establishing that the leachate was intermediate-aged, its characteristics were initially identified using physical, chemical and elemental analyses. Six parallel-connected electrode plates with stainless steel as the cathode and aluminium as the anode were used to construct an electrocoagulation cell. Under a variety of experimental conditions, including pH, temperature, agitation rate (AR), distance between electrodes (DBE), current density (CD), NaCl and Na_2_SO_4_ concentrations, and EC time, the turbidity, NH_3_-N and COD disposal efficiencies from leachate were simultaneously examined. This study additionally investigated the kinetics of turbidity, NH_3_-N and COD removal from the leachate and determined the operating costs for electrocoagulation.

## Material and methods

### Leachate features

Leachate from a landfill in Bingöl, Turkey, was used in this study. Home solid waste creation was a major factor in the landfill’s construction. The active area of the landfill is located 13 km from the city’s centre, toward the Bingöl-Muş Road. The structure’s architectural plan was separated into two portions, each with three stages. The daily capacity of the landfill is 831,950 m^3^. The towns of Solhan, Genç, Ilıcalar, Merkez, Sancak, Çaytepe, Arakonak and Servi are among those that use landfill. The foundation of the landfill is composed of a layer of synthetic geomembrane and a layer of mineral-containing impermeability. To drain the leachate at the top of the ground insulation, a drainage layer was built. At the leachate storage pool, surface waters from the surrounding area are gathered when it rains and are released into the environment via surface water–collecting ditches. A 20-L sample of landfill leachate was taken from the reservoir using a container made of high-density polyethylene. The leachate sample was immediately driven to the lab from the sanitary landfill.

The leachate was then filtered using a 0.45-mm pore filter to eliminate suspended particulate matter that would have inhibited turbidity, NH_3_-N and COD measurements. To analyse the physical and chemical characteristics of the leachate, the sample was maintained in a refrigerator at + 4 °C. The initial pH, turbidity, NH_3_-N and COD values of the landfill leachate sample were established in order to investigate turbidity, NH_3_-N and COD disposal efficiencies. The results of several chemical and physical tests performed on the leachate are displayed in Table 1. The leachate had values for pH, COD, NH_3_-N and turbidity of 8.35, 4175 mg of oxygen/L, 2438 mg/L and 228 NTU (or 285 mg of (NH_2_)_2_H_2_SO_4_/L), respectively, as given in Table 1.

**Table 1:** Some physical and chemical characteristics of the intermediate landfill leachate

Parameter	Unit	Value
pH		8.35
Conductivity	mS/cm	47
Total solids	mg/L	7700
Total volatile solids	mg/L	2871
Suspended solids	mg/L	221
Volatile suspended solids	mg/L	35
TDS	mg/L	6654
COD	mg/L	4175
BOD_5_	mg/L	1650
chloride	mg/L	2872
BOD_5_/COD	mg/L	0.4
NH_3_-N	mg/L	2438.7
Turbidity	NTU	228
Colour (RES)		
436 nm, R. Yellow RR Gran	cm^−1^	1063
525 nm, R. Red RR Gran	cm^−1^	576
620 nm, R. Blue RR Gran	cm^−1^	415
Li	ppm	0.604
B	ppm	4.954
Al	ppm	6.363
K	ppm	687.9
Ca	ppm	13.188
Ti	ppm	0.426
V	ppm	0.242
Cr	ppm	0.312
Mn	ppm	0.808
Fe	ppm	3.398
Co	ppm	0.106
Ni	ppm	0.03
Rb	ppm	0.436
Sr	ppm	1.053
Mo	ppm	0.012
Rh	ppm	0.18
Pd	ppm	2.907
Sec	ppm	0.007
Hf	ppm	0.222
Ta	ppm	1.538
W	ppm	0.261

For the electrocoagulation process, a 20 cm × 8 cm × 10 cm plexiglass reactor was used. The electrodes are organised in a monopolar parallel configuration with three anodes and three cathodes. Throughout the reaction time, it was thoroughly mixed with a Daihan brand HS-30D model mechanical mixer, which was submerged in the reactor from the top to provide perfect mixing. For electrochemical reactions, 400 mL of filtered and 1/4 diluted raw leachate samples were added to the reactor. In the experimental studies, the CD was 15 mA/cm^2^, 20 mA/cm^2^ and 25 mA/cm^2^; the pH was 3, 5, 7, 8.35 and 9.5; the temperature was 20 °C, 30 °C, 40 °C, 50 °C and 60 °C; the AR was 100 rpm, 200 rpm and 300 rpm; the DBE was 1 cm, 1.5 cm and 2 cm; and the electrolyte concentration for NaCl and Na_2_SO_4_ was 1 mM, 5 mM and 10 mM, respectively.

After sampling the top clear water at intervals of 1 min, 3 min, 5 min, 10 min, 15 min, 20 min, 25 min, 30 min, 35 min and 40 min, the samples were passed through a 45-µm filter paper to quantify the disposal efficiencies of COD, NH_3_-N and turbidity throughout the electrocoagulation process.

**Fig. 1:**
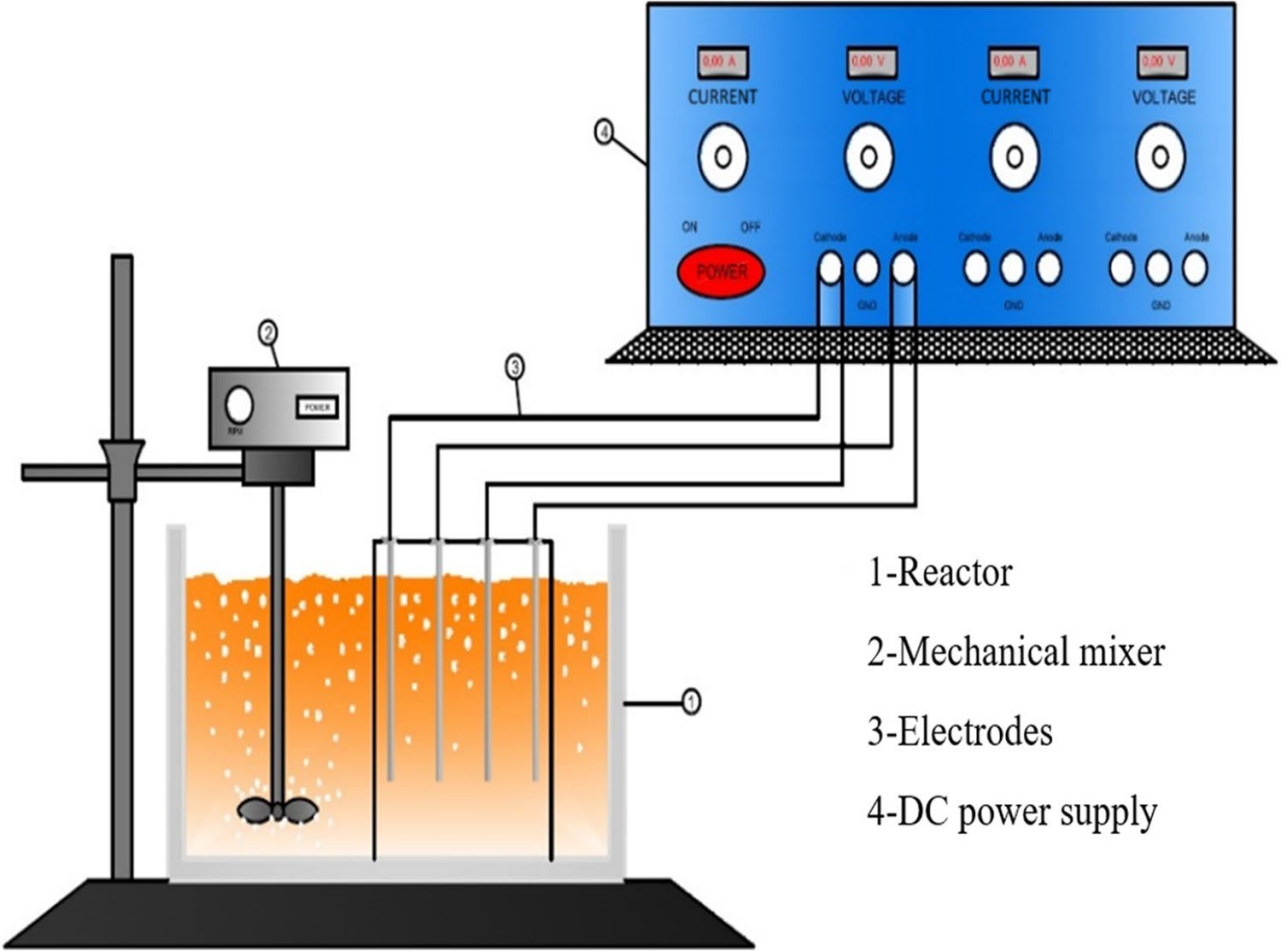
Schematic representation of the EC process

The anode and cathode materials were respectively made of aluminium and stainless steel. The plate electrodes used in the EC applications were 6 cm × 5 cm × 3 mm in size. Both electrodes were prepared for the experiments using fine sandpaper to clean them and then submerged them in a solution containing 1 M nitric acid. The electrodes were then dried at 105 °C after being washed with tap and distilled water (Myllymäki et al. 2018). The reactor and its characteristics are shown in Fig. 1 for the EC processes.

### Reactor cell used in electrocoagulation processes

Figure 1 depicts the configuration of the batch monopolar EC reactor employed in the current investigation. The EC cell has a capacity of 1 L and was constructed from plexiglass.

The electrode pairs (anode and cathode) were composed of six parallel pieces of aluminium and stainless steel plates with dimensions of 6 cm in length, 2 mm in depth and 5 cm in depth, with a surface area of 30 cm^2^. In the cell, the electrodes were installed on a plastic plate with perforations. Three anodes and three cathodes were employed in the batch monopolar electrochemical cell, which had six independent electrodes wired into the DC power source (AA Tech ADC-3303D, 0–3 A, 0–60 V). Three Al electrodes were attached to the power supply’s positive output as anodes. Three insoluble stainless steel electrodes were employed as cathodes and connected to the supply’s negative output. For each experiment, 400 mL of leachate was utilised.

### Chemicals

The chemicals used in this study were obtained from Sigma-Aldrich (USA) and Merck (USA). All chemicals were of analytical quality and employed exactly as they were obtained. Solutions made from sulphuric acid (H_2_SO_4_; Merck (2037554); purity 98.0%) and sodium hydroxide (NaOH; Sigma-Aldrich (57652336); purity ≥ 98.0%) were used to modify the pH levels of the leachate. COD values were calculated using solutions derived from H_2_SO_4_ (Merck (2037554); purity 98.0%), potassium dichromate (K_2_Cr_2_O_7_; Sigma-Aldrich (207802); purity ≥ 99.0%) and silver sulphate (Ag_2_SO_4_; Sigma-Aldrich (497266); purity ≥ 99.99%).

Analyses of NH_3_-N were performed using solutions prepared from phenol (C_6_H_5_OH; Sigma-Aldrich (16016); purity 99.5%), sodium nitroprusside dihydrate (Na_2_[Fe(CN)_5_NO]·2H_2_O; Sigma-Aldrich (71778); purity ≥ 99%), trisodium citrate dihydrate (Na_3_C_6_H_5_O_7_·2H_2_O; Merck (6104939); purity ≥ 99%), NaOH (Sigma-Aldrich (57652336); purity ≥ 98.0%) and sodium hypochlorite (NaOCl; Sigma-Aldrich (71696); purity ~ 10%).

Turbidity values were determined using solutions derived from hydrazine sulphate salt (NH_2_NH_2_·H_2_SO_4_; Sigma-Aldrich (216046); purity ≥ 99.0%) and hexamethylenetetramine (C_6_H_12_N_4_; Sigma-Aldrich (398160); purity ≥ 99.0%).

The chloride content in the leachate was determined using solutions prepared from hydrogen peroxide (H_2_O_2_; Sigma-Aldrich (H1009); purity 30% (w/w) in H_2_O), aluminium hydroxide (Al(OH)_3_; Sigma-Aldrich (239186); purity 100%), potassium chromate (K_2_CrO_4_; Sigma-Aldrich (12249); purity ≥ 99.0%) and silver nitrate (AgNO_3_; Sigma-Aldrich (209139); purity ≥ 99.0%).

### Analyses

#### Physical analyses

The conductivity, pH and total dissolved solid (TDS) parameters in the leachate have been examined using Thermo Scientific Orion Versa Star benchtop multiparameter equipment. Standard methods were applied to determine the turbidity, colour and total suspended particles in the leachate (Rice et al. 2012).

#### Chemical analyses

A reference method known as the SM 5220.D closed reflux colorimetric approach was employed in the COD analysis. At a wavelength of 600 nm, COD concentrations were calculated using the absorbance readings (Rice et al. 2012). NH_3_-N was calculated using the standard 4500-F phenate approach. The sample concentration was established using an equation from absorbance values versus concentration values at 640 nm (Rice et al. 2012). The chloride analysis was performed via the standard 4500-CI-B argentometric technique. By deducting the sample volume from the blank sample volume, the sample concentration was calculated (Rice et al. 2012).

COD, NH_3_-N and turbidity analyses were repeated three times in this study; the arithmetic mean of results was taken; and standard deviations were displayed in all graphs.

#### Biological analysis

The BOD_5_ content was measured using an Oxi 700 instrument from the Orbeco brand.

#### Elemental analyses

The ICP-MS NexION 2000 C gear from PerkinElmer, Inc. (USA), was used to perform research on the leachate’s heavy metal ions.

### Kinetic investigations for the EC process

Pseudo-first-order (PFO) and pseudo-second-order (PSO) equations were employed to determine reaction rates and rate constants for COD, NH_3_-N and turbidity removal from the leachate under various experimental conditions. The PFO reaction rate equation was employed to determine the reaction rate constants since the correlation coefficients for PFO reaction rates under various experimental settings were greater than those for PSO. Due to the poor correlation coefficients, the graphs related to the PSO rate equation were not included in the paper. Depending on the applied CD, the pollutant concentration (such as COD, NH_3_-N and turbidity) and the amount of matching metal hydroxides generated determine the efficacy rate of the EC process (Nandi and Patel 2013). Therefore, the following reaction kinetics was used to explain the rate of COD, NH_3_-N and turbidity disposal:

1 $$-\frac{{\text{d}}C}{{\text{d}}t}=k \left[{C}_{{\text{Al}}}\right]\left[C\right]$$

2 $$-\frac{{\text{d}}C}{{\text{d}}t}={k}^{\mathrm{^{\prime}}}\times C$$

where *C* is the concentration of COD, NH_3_-N and turbidity in the leachate; *C*_Al_ is the concentration of aluminium hydroxide in the solution (mg/L), which is assumed to be constant for a given current density; and *k* is the COD, NH_3_-N and turbidity removal rate constant. *C*_Al_ and *k* are constants in Eq. (1), and their product is therefore a constant. Hence, *k* × *C*_Al_ appears to be a constant. Equation (1) is equal to Eq. (2), and the *k*′ constant is used to signify this product. Thus, *k*′ is a PFO rate constant (min^−1^).

If Eq. (2) is integrated at the conditions *t* = 0, *C* = *C*_0_ and *t* = *t* and *C* = *C*, the linear form of Eq. (2) is obtained.

3 $${\text{ln}}\left(\frac{{C}_{0}}{C}\right)={k}^{\mathrm{^{\prime}}}t$$

In Eq. (3), if ln(*C*_0_/*C*) values are plotted against *t* values, *k*′ is determined by the slope of the line.

### EC process operating costs

Using the following equations, the results of the EC process were assessed. Initial and final COD concentrations (*C*_0_ and *C*, mg oxygen (O_2_)/L) were used to calculate the percentage of COD removal (*Y*, %).

4 $$Y\mathrm{\%}= \frac{({C}_{0}- C)}{{C}_{0}}\times 100$$

The following equations were used to calculate the total amount of anodes that would dissolve into a solution during an electrocoagulation process in accordance with Faraday’s law.

5 $${C}_{{\text{energy}}}=\frac{U\times I\times t}{V}$$

Equation (6) was used to determine electrode usage.

In this equation, *C*_energy_ represents the energy consumption (kWh/m^3^), *U* for voltage, *I* for current intensity (amperes), *t* for time (hours) and *V* for leachate volume (m^3^).

6 $${C}_{{\text{electrode}}}=\frac{i\times t\times {M}_{{\text{w}}}}{z\times F\times V}$$

In Eq. (6), *M*_w_ denotes the mass weight of the anode that was utilised (*M*_w_, Al = 26.98 g/mol), *z* denotes the anode’s chemical equivalent or the number of electrons exchanged during the reaction (*z*_Al_ = 3) and *F* denotes the Faraday constant (96,487 C/mol *e*^−^). *C*_electrode_ (kg/m^3^) is the quantity of dissolved aluminium metal electrode in the reactor (measured in kilogrammes per cubic meter).

Equation (7) was used to calculate the EC process’s energy depletion per unit of time.

7 $${\text{UED}}=\frac{I\times {\int }_{0}^{t}U\times {\text{d}}t}{1000\times V\times {C}_{0}\times \left({Y}_{t}|100\right)}$$

In Eq. (7), UED denotes unit energy demand (kWh/kg COD), *U* denotes voltage (volts), *I* denotes current intensity (ampere), *t* denotes time (hour), *V* denotes leachate volume (m^3^), *C*_0_ denotes initial COD concentration (kg/m^3^) and *Y*_*t*_ denotes removal efficiency (%) at time *t*.

Equation (8) calculates the electrical consumption cost by considering the electrical energy and the used anode material.

8 $${\text{EOC}}={\text{EEC}}+{\text{MC}}={\text{UED}}\times {\text{EEP}}+{\text{MC}}$$

where EOC is the electrical operating cost ($/kg COD), EEC is the electrical energy consumption ($/kg COD), MC is the cost of electrode material consumed ($/kg COD), UED is the unit energy demand ($/kWh) and EEP is the electrical energy price ($/kWh).

9 $${\text{MC}}=\frac{I\times t\times A}{n\times F}\times \frac{{\text{AMP}}}{V\times {C}_{0}\times \left({Y}_{t}|100\right)}$$

The anode material price in Eq. (6) is AMP ($/kg).

## Results and discussion

### *The influence of CD on the disposal efficiency of turbidity, NH*_*3*_*-N and COD*

**Fig. 2:**
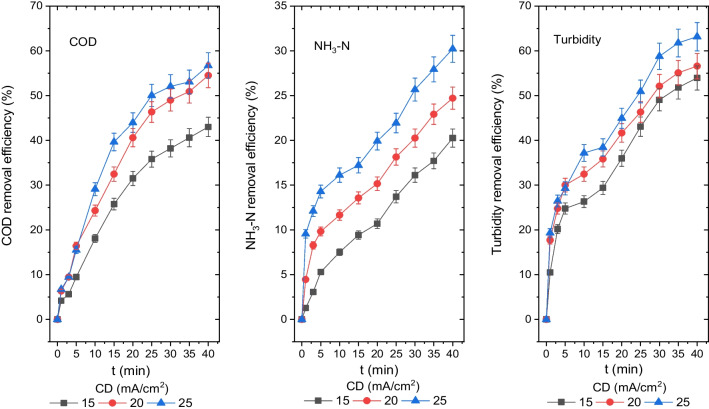
Disposal efficiencies of COD, NH_3_-N and turbidity from the leachate at different CDs (COD 1044 mg O_2_/L, NH_3_-N 204 mg/L, turbidity 71.3 mg (NH_2_)_2_H_2_SO_4_/L, *T* 20°, pH 8.35, AR 300 rpm, electrolyte concentration 0 and DBE 1 cm)

The CD is a crucial factor in the EC process for coagulant dose in turbidity, NH_3_-N and COD removal from leachate (Li et al. 2011). In Fig. 2, it is represented how COD, NH_3_-N and turbidity removals from leachate were affected by CD of 15 mA/cm^2^, 20 mA/cm^2^ and 25 mA/cm^2^, respectively. The values of COD, NH_3_-N, turbidity, BOD_5_ and BOD_5_/COD were calculated after the reactor had undergone a reaction for 40 min. After the EC process of 40 min for 15 mA/cm^2^, 20 mA/cm^2^ and 25 mA/cm^2^ CD, the effluent pH values were determined to be 8.97, 9.36 and 9.83, respectively.

After a 40-min reaction for CD of 15 mA/cm^2^, 20 mA/cm^2^ and 25 mA/cm^2^, the concentrations of COD, NH_3_-N and turbidity were found to be 595 mg O_2_/L, 475 mg O_2_/L and 452 mg O_2_/L; 163 mg/L, 153 mg/L and 142 mg/L; and 33 mg (NH_2_)_2_H_2_SO_4_/L, 31 mg (NH_2_)_2_H_2_SO_4_/L and 26 mg (NH_2_)_2_H_2_SO_4_/L, and their disposal efficiencies were found to be 43%, 55% and 57%; 20%, 25% and 30%; and 54%, 56% and 63%, respectively. The optimal disposal efficiencies of COD, NH_3_-N and turbidity were determined as 57%, 30% and 63%, respectively, for a CD of 25 mA/cm^2^.

According to Fig. 2, when CD increased, the efficiency of removing COD, NH_3_-N and turbidity also increased. Also, during a 40-min reaction at CD of 15 mA/cm^2^, 20 mA/cm^2^ and 25 mA/cm^2^, BOD_5_ concentrations and BOD_5_/COD ratios were calculated to be 144 mg O_2_/L, 208 mg O_2_/L and 296 mg O_2_/L and 0.24, 0.46 and 0.66, respectively. It can be stated that the BOD_5_/COD ratios for CD of 20 mA/cm^2^ and 25 mA/cm^2^ are in the range of 0.4 and 0.8 standard limit values, respectively, for untreated wastewater by Turkish regulations. Therefore, this method is suitable for discharging treated effluent into sewage systems.

The amount of coagulant formed by the aluminium anode’s dissolution and the frequency and size of bubbles are all influenced by the CD (Khosla et al. 1991; Holt et al. 2002). Thus, the CD significantly influenced the disposal of pollutants, including COD, NH_3_-N and turbidity from the leachate. The improvement in COD, NH_3_-N and turbidity removal efficiency is explained by an increase in coagulant and bubble formation rates.

Thus, faster and more effective pollution removal was achieved by the increase in CD. The amount of aluminium and hydroxide ions produced in the electrocoagulation cell at any given time is related to Faraday’s equation (Eq. 10).

10 $$m=\frac{ItM}{zF}$$

where *I* is the current intensity, *z* is the number of electrons exchanged during the reaction, *t* is the period, *M* is the molecular weight of the aluminium or hydroxide ion (g mol^−1^) and *F* is the Faraday constant (96486 C mol^−1^). The Al(OH)_3_ solid forms more quickly and in larger quantities in a solution because the dissolving potential of aluminium in the anode rises with the augmentation in CD, as given by Eq. (10). The formed Al(OH)_3_ solid is thought to interact with the dissolved components in the solution environment, wrap the colloidal structures in the form of a network and remove them from the solution environment.

In addition, when the CD increased, so did the rate of the formation of H_2_ gas bubbles, while the size of the H_2_ bubbles dropped. These two variables helped the flotation of pollutants with H_2_ gas, increasing the disposal efficiencies for turbidity, NH_3_-N and COD. The rise in pH values in the solution was also influenced by the augmentation of CD during NH_3_-N removal from the leachate. The NH_3_-N is thought to be removed from the environment through the H_2_ gas bubbles and the air transferred to the solution through mixing.

### *The influence of initial pH on the disposal efficiency of turbidity, NH*_*3*_*-N and COD*

**Fig. 3:**
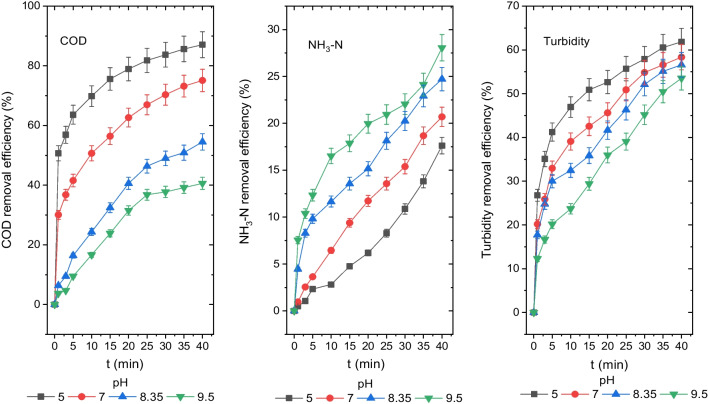
Disposal efficiencies of COD, NH_3_-N and turbidity from the leachate at different initial pH values (COD 1044 mg O_2_/L, NH_3_-N 204 mg/L, turbidity 71.3 mg (NH_2_)_2_H_2_SO_4_/L, CD 20 mA/cm.^2^, *T* 20 °C, AR 300 rpm, electrolyte concentration 0 and DBE 1 cm)

In the EC process, it is well known that the pH of the sample has a significant impact on the disposal of turbidity, NH_3_-N and COD from leachate (Chen et al. 2000). In removing turbidity, NH_3_-N and COD from the leachate, the effects of initial pH values of 5, 7, 8.4 and 9.5 were looked into and are shown in Fig. 3. The effluent pH values were determined as 7.8, 8.8, 9.2 and 10.1 for the initial pH values of 5, 7, 8.35 and 9.5, respectively.

The pH values of the electrocoagulated leachate were reported to rise in the last stage of process due to an increase in OH^−^ ions produced by the reduction of water on the cathode electrode surface (Aswathy et al. 2016). Equation (11) shows this reaction.

11 $${2{\text{H}}}_{2}{\text{O}}+2{{\text{e}}}^{-}\to {{\text{H}}}_{2}+{2{\text{OH}}}^{-}$$

After a 40-min reaction for pH values of 5, 7, 8.35 and 9.5, the concentrations of COD, NH_3_-N and turbidity were found to be 135 mg O_2_/L, 260 mg O_2_/L, 475 mg O_2_/L and 620 mg O_2_/L; 168 mg/L, 162 mg/L, 153 mg/L and 146 mg/L; and 27 mg (NH_2_)_2_H_2_SO_4_/L, 30 mg (NH_2_)_2_H_2_SO_4_/L, 31 mg (NH_2_)_2_H_2_SO_4_/L and 33 mg (NH_2_)_2_H_2_SO_4_/L, and their disposal efficiencies were found to be 87%, 75%, 55% and 41%; 18%, 21%, 25% and 28%; and 62%, 58%, 57% and 54%, respectively.

At pH 5, pH 9.5 and pH 5 values, the optimum disposal efficiencies of COD, NH_3_-N and turbidity were determined as 87%, 28% and 62%, respectively. As the initial pH value increased, the turbidity and COD disposal efficiency decreased as seen in Fig. 3. When pH levels rose, so did the efficiency of NH_3_-N removal. Also, at pH 5 and 8.35 values, BOD_5_ concentrations and BOD_5_/COD ratios were found to be 85.75 mg O_2_/L and 208 mg O_2_/L and 0.64 and 0.44, respectively, following a reaction of 40 min. For untreated wastewater by Turkish regulations, BOD_5_/COD ratios for the effluent at pH 5 and 8.35 are between 0.4 and 0.8 standard limit values. Therefore, electrocoagulated leachate can be disposed of in sewage systems. At the pH level of 5, the optimum COD and turbidity removal efficiency were attained. The impact of pH value on COD, NH_3_-N and turbidity removal from the leachate was explained using a diagram of aluminium species hydrolysed in deionized water (Elkins and Nelson 2002). Aluminium hydrolyses at pH 5–7 to form the ions Al^3+^, Al(OH)^2+^ and Al(OH)^+^, which are effective for destabilising colloidal structures. These structures precipitate destabilised colloidal materials by wrapping them in a network in the form of mainly solid sludge Al(OH)_3_ (Marañón et al. 2008; Mahmad et al. 2016). As a result, COD and turbidity removal efficiencies rose with lowering pH levels. The hydrolysed alum solid sludge is present at pH 8.35 and pH 9.5 as Al(OH)_3_ and Al(OH)^4−^ ions, respectively. The efficiency of COD and turbidity removal was reduced by the production of negatively charged Al(OH)^4−^ monopolymers at high pH levels (Akyol 2012).

The initial pH values of 9.5 and 8.35 lead to the highest NH_3_-N removal efficiency from the leachate. The NH_3_-N is said to move away from the alkaline environment due to interactions between the NH_3_-N and the Al(OH)_3_ solid, the H_2_ gas bubbles that form at the cathode and the air that is provided to the solution. It may be said that NH_3_-N is adsorbed onto Al^3+^, Al(OH)^2+^ and Al(OH)^+^ ions as well as Al(OH)_3_ solids in the solution medium at pH 5 and pH 7 values. As shown in Fig. 3, when the pH value declined, the efficiency of NH_3_-N removal also reduced.

### *The influence of initial temperature on the disposal efficiency of turbidity, NH*_*3*_*-N and COD*

**Fig. 4:**
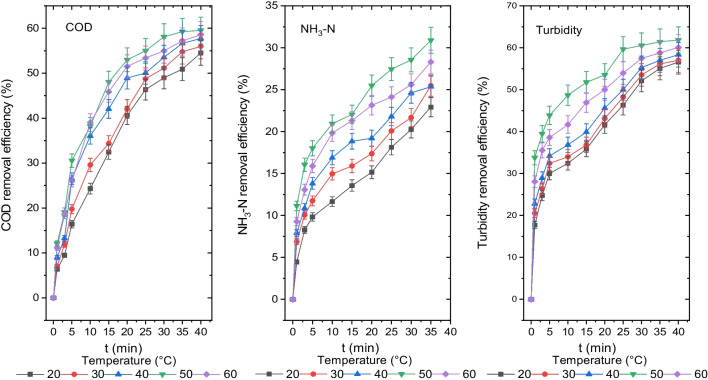
Disposal efficiencies of COD, NH_3_-N and turbidity from the leachate at different initial temperature values (COD 1044 mg O_2_/L, NH_3_-N 204 mg/L, turbidity 71.3 mg (NH_2_)_2_H_2_SO_4_/L, CD 20 mA/cm.^2^, pH 8.35, AR 300 rpm, electrolyte concentration 0 and DBE 1 cm)

Even though electrocoagulation has been used for over a century, the effect of temperature on this process has attracted little consideration (Chen 2004). The findings for the disposal of turbidity, NH_3_-N and COD at starting temperatures of 20 °C, 30 °C, 40 °C, 50 °C and 60 °C are displayed in Fig. 4. At initial temperature values of 20 °C, 30 °C, 40 °C, 50 °C and 60 °C, the COD, NH_3_-N and turbidity concentrations were 475 mg O_2_/L, 460 mg O_2_/L, 441 mg O_2_/L, 412 mg O_2_/L and 432 mg O_2_/L; 157 mg/L, 152 mg/L, 147 mg/L, 141 mg/L and 147 mg/L; and 31 mg (NH_2_)_2_H_2_SO_4_/L, 30 mg (NH_2_)_2_H_2_SO_4_/L, 29 mg (NH_2_)_2_H_2_SO_4_/L, 27 mg (NH_2_)_2_H_2_SO_4_/L and 28 mg (NH_2_)_2_H_2_SO_4_/L, and their removal efficiencies were 55%, 56%, 58%, 60% and 59%; 23%, 25%, 26%, 31% and 28%; and 56%, 57%, 58%, 62% and 60%, respectively.

The optimum disposal efficiencies for COD, NH_3_-N and turbidity were defined to be 60%, 31% and 62%, respectively, at 50 °C. The disposal of turbidity, NH_3_-N and COD was more effective at higher temperatures, as shown in Fig. 4, which ranged from 20 to 50 °C. Even so, it was determined that the disintegration of the flocs as the initial temperature value increased from 50 to 60 °C caused a reduction in the COD, NH_3_-N and turbidity removal efficiency.

Similar to other chemical reaction rates, the electrochemical reaction rate rises as the temperature of the solution increases (Modirshahla et al. 2008). The following explanation reveals why leachate wastewater treatment efficiency of COD, NH_3_-N and turbidity increases as temperature rises (Al-Raad et al. 2020):

1. The dissolution of Al^3+^ ions in the aluminium anode is accelerated and increased by raising the solution temperature, which also causes the formation of Al^3+^ monopolymers and Al(OH)_3_ solid.
2. According to the Stokes–Einstein equation, increasing the temperature increases the diffusivity of aluminium into the bulk solution, which raises the rate at which aluminium is transferred from the anode surface to the solution mass.

These components have positively influenced the COD, NH_3_-N and turbidity removal efficiency from the leachate. A rise in the initial temperature of the leachate and the ammonia’s reduced solubility with temperature are two more factors that contribute to the disposal efficiency of NH_3_-N.

### *The influence of the DBE on the disposal efficiency of turbidity, NH*_*3*_*-N and COD*

**Fig. 5:**
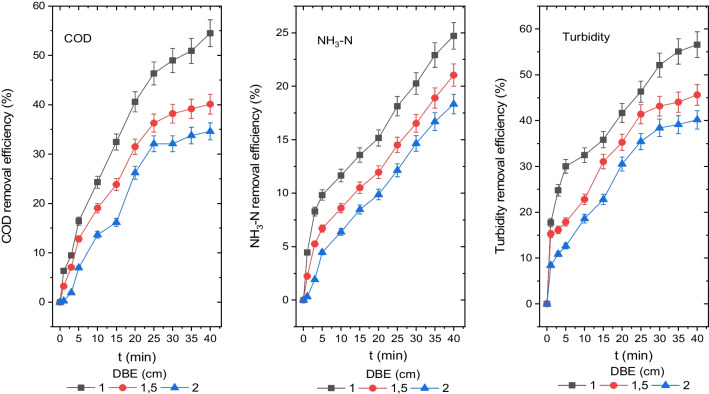
Disposal efficiencies of the COD, NH_3_-N and turbidity from the leachate depending on the DBE (COD 1044 mg O_2_/L, NH_3_-N 204 mg/L, turbidity 71.3 mg (NH_2_)_2_H_2_SO_4_/L, CD 20 mA/cm.^2^, pH 8.35, *T* 20 °C, AR 300 rpm and electrolyte concentration 0)

The results of removing COD, NH_3_-N and turbidity from leachate at distances of 1 cm, 1.5 cm and 2 cm between the electrodes are shown in Fig. 5. After a reaction lasting 40 min, concentrations for COD, NH_3_-N and turbidity were calculated as 475 mg O_2_/L, 625 mg O_2_/L and 683 mg O_2_/L; 153 mg/L, 161 mg/L and 166 mg/L; and 31 mg (NH_2_)_2_H_2_SO_4_/L, 39 mg (NH_2_)_2_H_2_SO_4_/L and 43 mg (NH_2_)_2_H_2_SO_4_/L, and their removal efficiencies were calculated as 55%, 40% and 35%; 25%, 21% and 18%; and 57%, 46% and 40%, for DBE of 1 cm, 1.5 cm and 2 cm, respectively.

The optimum COD, NH_3_-N and turbidity disposal efficiencies were determined to be 55%, 25% and 57%, respectively, for an electrode distance of 1 cm. According to Fig. 5, the efficiency of turbidity, NH_3_-N and COD disposal reduced as the distance between the aluminium electrodes increased.

An essential factor for the performance of the EC process is the impact of the DBE. According to the literature, a variety of variables such as electrode structure, hydrodynamic conditions and pollutant type influence how the DBE contributes to the electrocoagulation process (Daneshvar et al. 2004; Modirshahla et al. 2007). When the DBE is increased, the electrocoagulation process’s ability to remove COD, NH_3_-N and turbidity decreases, which is caused by the solution’s decreased electrical conductivity.

The decrease in electrical conductivity also reduces the dissolution of the ion metal, minimizing the number of the flock in the bulk solution. As the solution’s resistance increases, a high voltage must be given to the electrodes while removing COD, NH_3_-N and turbidity through a large gap between the electrodes. This is necessary to maintain EC process’s performance. The expense of the treatment process rises as a result of the usage of high voltage throughout the process. In addition, increasing DBE inhibits the interaction of colloidal and dissolved components in the leachate with hydroxyl, ionic aluminium species and aluminium hydroxide solids (Khandegar and Saroha 2012; Naje et al. 2019; Al-Raad et al. 2020). As a result of these factors, the efficiency of turbidity, NH_3_-N and COD disposal reduced as the DBE increased.

### *The influence of NaCl support electrolyte on the disposal efficiency of turbidity, NH*_*3*_*-N and COD*

**Fig. 6:**
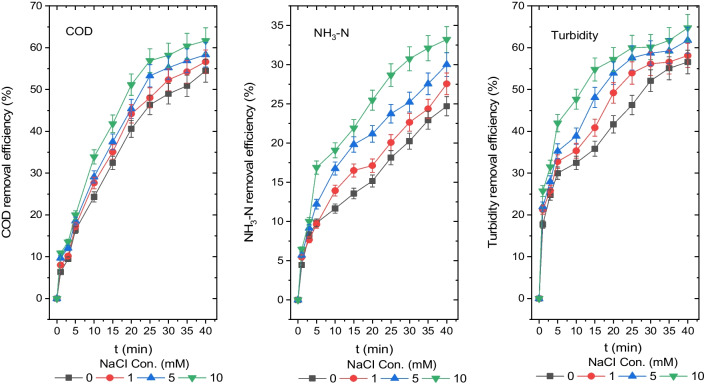
Disposal efficiencies of COD, NH_3_-N and turbidity from the leachate depending on different NaCl supporting electrolyte concentrations (COD 1044 mg O_2_/L, NH_3_-N 204 mg/L, turbidity 71.3 mg (NH_2_)_2_H_2_SO_4_/L, *T* 20 °C, pH 8.35, AR 300 rpm and DBE 1 cm)

The electrical conductivity of the solution is an important variable in electrical energy savings. To improve the electrical conductivity of the leachate, NaCl was employed as a supporting electrolyte. The impact of NaCl concentrations of 0 mM, 1 mM, 5 mM and 10 mM on reducing COD, NH_3_-N and turbidity from the leachate was examined and is illustrated in Fig. 6. As a consequence of a 40-min reaction of the leachate with an initial natural pH value of 8.35, the effluent pH values were determined to be 9.27, 9.33 and 9.47, respectively, for NaCl concentration values of 0 mM, 1 mM, 5 mM and 10 mM.

COD, NH_3_-N and turbidity concentrations from the leachate after a 40-min reaction were 475 mg O_2_/L, 453 mg O_2_/L, 435 mg O_2_/L and 400 mg O_2_/L; 153 mg/L, 148 mg/L, 143 mg/L and 136 mg/L; and 31 mg (NH_2_)_2_H_2_SO_4_/L, 30 mg (NH_2_)_2_H_2_SO_4_/L, 27 mg (NH_2_)_2_H_2_SO_4_/L and 25 mg (NH_2_)_2_H_2_SO_4_/L, and their removal efficiencies were 55%, 57%, 59% and 62%; 25%, 27%, 30% and 33%; and 56%, 58%, 62% and 65%, respectively, for NaCl concentrations of 0 mM, 1 mM, 5 mM and 10 mM. The COD, NH_3_-N and turbidity removal efficiency improved with the concentration of the support electrolyte NaCl, as shown in Fig. 6.

Furthermore, after a 40-min reaction using 0 mM NaCl and 10 mM NaCl concentrations, the BOD_5_ concentrations and BOD_5_/COD ratios were identified as 208 mg O_2_/L and 238 mg O_2_/L and 0.44 and 0.6, respectively. The BOD_5_/COD ratios for the effluent at 0 mM NaCl and 10 mM NaCl concentrations were between 0.4 and 0.8, which are typical limit values for untreated wastewater in Turkey.

The passivation of the anode electrode decreases as the Cl^−^ ion concentration in the solution increases, and its capacity to conduct electricity improves. Cl^−^ ions are released at the anode during the EC process to generate Cl_2_ gas, which is subsequently chemically transformed into ClO^−^ ion, which may efficiently oxidise pollutants (Wang et al. 2009).

As a result, the adsorption of monomers is caused by intermediate products generated by the oxidation of dissolved compounds in solution. Moreover, by enveloping destabilised colloids and oxidised intermediates in a network, the Al(OH)_3_ solid produces precipitation. Thus, COD and turbidity removal efficiency rose when Cl^−^ ions increased.

The ammonia type predominates in alkaline surroundings. Ammonia was eliminated from the surroundings by direct oxidation to nitrogen or through the air stripping process, which produced extremely tiny bubbles as a result of electrolysis. The presence of chlorine in the environment aids in the decomposition of ammonia via the indirect oxidation process. An indirect process removes ammonia at a high level. Because of the creation of hypochlorite ions or hypochlorous acid as a result of the reaction of CI^−^ ions at the anode, the ammonia removal efficiency improved owing to the indirect conversion of ammonia into nitrogen gas. In neutral, alkaline and acidic conditions, the mechanisms of indirect electro-oxidation and ammonia removal differ. Ammonia removal mechanism in alkaline media includes adsorption/oxidation (direct route), oxidation with chlorine and air stripping. At pH 10, the most common ammonia nitrogen and chlorine forms in the bulk solution are NH_3_ and OCl^−^, respectively. The equations are as follows:

12 $${2{\text{CI}}}^{-}+{2{\text{H}}}^{+}\to {{\text{CI}}}_{2}+{{\text{H}}}_{2}$$

13 $${{\text{CI}}}_{2}+{{\text{H}}}_{2}{\text{O}}\leftrightarrow {\text{HOCI}}+{{\text{H}}}^{+}+{{\text{CI}}}^{-}$$

14 $${\text{HOCI}}\leftrightarrow {{\text{OCI}}}^{-}+{{\text{H}}}^{+}$$

15 $${{\text{OCI}}}^{-}+{{\text{NH}}}_{3}\to {{\text{NH}}}_{2}{\text{CI}}+{{\text{OH}}}^{-}$$

Chloramine synthesis generates OH^−^ ions, according to Eq. (15). As a result of this process, the pH of the solution increases. Dichloramine is formed as a consequence of the reaction between NH_2_Cl and OCI^−^ (Karukstis and Van Hecke 2003).

16 $${{\text{OCI}}}^{-}+{{\text{NH}}}_{2}{\text{CI}}\to {{\text{NHCI}}}_{2}+{{\text{OH}}}^{-}$$

Morris and Wei (1969) developed a process for the breakdown of dichloramine using an OH^−^ ion catalyst in an alkaline media (Weber Jr and Morris 1963).

17 $${{\text{NHCI}}}_{2}+{{\text{H}}}_{2}{\text{O}}\to {\text{NOH}}+{2{\text{H}}}^{+}+{2{\text{CI}}}^{-}$$

This reaction occurs as follows to produce the final products:

18 $${\text{NOH}}+{{\text{NH}}}_{2}{\text{CI}}\to {{\text{N}}}_{2}+{{\text{H}}}_{2}{\text{O}}+{{\text{H}}}^{+}+{{\text{CI}}}^{-}$$

19 $${\text{NOH}}+{2{\text{OCI}}}^{-}\to {{\text{NO}}}_{3}^{-}+{{\text{H}}}^{+}+{2{\text{CI}}}^{-}$$

### *The influence of Na*_*2*_*SO*_*4*_* support electrolyte on the disposal efficiency of turbidity, NH*_*3*_*-N and COD*

**Fig. 7:**
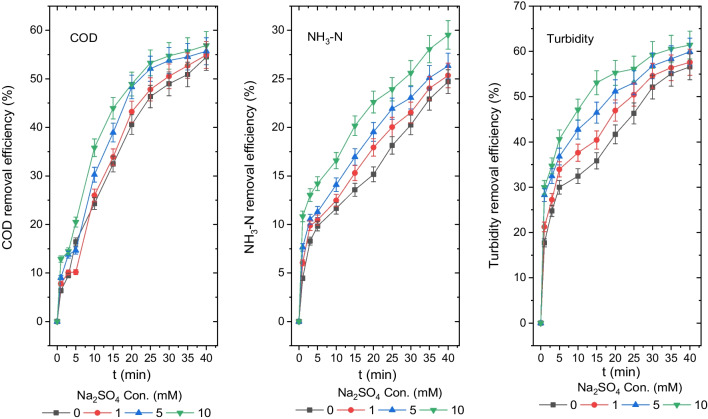
Disposal efficiencies of COD, NH_3_-N and turbidity from the leachate at different Na_2_SO_4_ concentrations (COD 1044 mg O_2_/L, NH_3_-N 204 mg/L, turbidity 71.3 mg (NH_2_)_2_H_2_SO_4_/L, *T* 20 °C, pH 8.35, AR 300 rpm and DBE 1 cm)

The solution’s electrical conductivity is critical for transporting ions dissolved at the anode to the bulk solution and saving electrical energy. To improve the electrical conductivity of the solution, Na_2_SO_4_ was employed as a supporting electrolyte. The impact of Na_2_SO_4_ concentrations of 0 mM, 1 mM, 5 mM and 10 mM on removing COD, NH_3_-N and turbidity from the leachate was examined in detail and is shown in Fig. 7.

As a consequence of a 40-min reaction of the leachate with an initial natural pH value of 8.35, the effluent pH values were determined to be 9.04, 9.15 and 9.24, respectively, for Na_2_SO_4_ concentration values of 0 mM, 1 mM, 5 mM and 10 mM. After 40 min of reaction time for Na_2_SO_4_ concentrations of 0 mM, 1 mM, 5 mM and 10 mM, the COD, NH_3_-N and turbidity concentrations were 475 mg O_2_/L, 471 mg O_2_/L, 463 mg O_2_/L and 450 mg O_2_/L; 153 mg/L, 152 mg/L, 150 mg/L and 144 mg/L; and 31 mg (NH_2_)_2_H_2_SO_4_/L, 30 mg (NH_2_)_2_H_2_SO_4_/L, 29 mg (NH_2_)_2_H_2_SO_4_/L and 28 mg (NH_2_)_2_H_2_SO_4_/L, and their disposal efficiencies were 54.5%, 54.9%, 55.7% and 56.8%; 24.7%, 25.4%, 26.3% and 29.5%; and 56.6%, 57.7%, 59.9% and 61.4%, respectively. At 10 mM Na_2_SO_4_, the highest COD, NH_3_-N and turbidity disposal efficiencies were identified as 56.8%, 29.5% and 61.4%, respectively. The turbidity, NH_3_-N and COD disposal efficiencies were partially enhanced by Na_2_SO_4_ support electrolyte, as illustrated in Fig. 7.

The following hypotheses were suggested as to why there is not much of improvement in removal efficiency in the presence of SO_4_^2−^ support ions in the solution: Ca^2+^, Mg^2+^ and CO_3_^2−^ are present in complex leachates. The CO_3_^2−^ and SO_4_^2−^ ions in the leachate are adsorbed on the aluminium anode in a CaCO_3_, MgCO_3_, CaSO_4_ and MgSO_4_ structure (Chou et al. 2009; Mansoorian et al. 2012). Moreover, dissolved O_2_ generated at the anode by the reduction of OH^−^ ions reacts with solid aluminium (Al°), generating an Al_2_O_3_ structure and became adsorbed on the anode surface (Mansoorian et al. 2012). The passivation of the aluminium electrode is caused by the adsorption of these structures on the anode surface. These adsorbed layers function as an insulator, increasing the ohmic resistance of the electrochemical cell and decreasing the disposal efficiencies of COD, NH_3_-N and turbidity. If CI^−^ ions are present in the solution medium in the leachate, these layers on the anode surface will react with them. The passivation layer is removed by CI^−^ ions, and these structures are transferred to the bulk solution as CaCl_2_, MgCl_2_ and AICI_3_. Thus, the concentration of aluminium species in the solution medium is enhanced. This investigation revealed that the leachate contained 81.6 mM chloride ion. As a result, SO_4_^2−^ ions in the presence of CI^−^ ions enhanced the efficiency of turbidity, NH_3_-N and COD disposal, by transporting the Al^3+^ ions at the anode to the bulk solution and improving conductivity.

### *The influence of AR on the disposal efficiency of turbidity, NH*_*3*_*-N and COD*

**Fig. 8:**
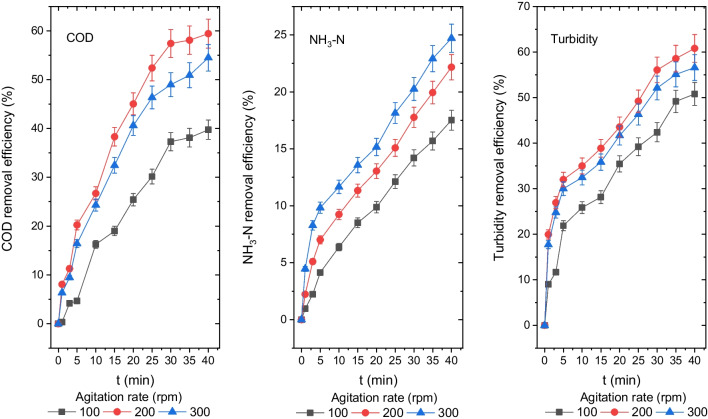
Disposal efficiencies of COD, NH_3_-N and turbidity from the leachate at different ARs (COD 1044 mg O_2_/L, NH_3_-N 204 mg/L, turbidity 71.3 mg (NH_2_)_2_H_2_SO_4_/L, CD 20 mA/cm.^2^, pH 8.35, *T* 20 °C, electrolyte concentration 0 mg/L and DBE 1 cm)

As illustrated in Fig. 8, ARs of 100 rpm, 200 rpm and 300 rpm were used to assess turbidity, NH_3_-N and COD disposal efficiency from the leachate. After a 40-min reaction at ARs of 100 rpm, 200 rpm and 300 rpm, the concentrations for COD, NH_3_-N and turbidity were reported as 629 mg O_2_/L, 424 mg O_2_/L and 475 mg O_2_/L; 168 mg/L, 159 mg/L and 153 mg/L; and 35 mg (NH_2_)_2_H_2_SO_4_/L, 28 mg (NH_2_)_2_H_2_SO_4_/L and 31 mg (NH_2_)_2_H_2_SO_4_/L, and their disposal efficiencies were 40%, 59% and 55%; 14%, 18% and 21%; and 43%, 56% and 52%, respectively.

As the AR rose from 100 to 200 rpm, the efficiency of COD and turbidity disposal from leachate is enhanced. But, when AR increased from 200 to 300 rpm, COD and turbidity removal efficiency declined.

The NH_3_-N removal efficiency from the leachate augmented as AR augmented from 100 to 300 rpm, and the 300 rpm provided the best NH_3_-N disposal efficiency.

The EC process is believed to have included three stages (Bouhezila et al. 2011):

1. Synthesis of coagulates as a result of sacrificial electrode oxidation
2. Destabilisation of pollutant and particle suspensions
3. Aggregation of destabilised phases to produce flocs

A homogenous solution environment is believed to be crucial in the second step to ensure interaction between coagulants and pollutants. The optimum AR promotes anode dissolution by lowering anode passivity and transferring the coagulant to the bulk solution. Moreover, an appropriate AR homogenises the impurities and coagulants in the solution. Adsorption of colloids by coagulants at optimum AR and wrapping the destabilised structures in a net form by the Al(OH)_3_ solid produced in the solution enhanced COD and turbidity removal efficiencies with increasing AR from 100 to 200 rpm. The COD and turbidity disposal efficiencies decreased as the AR augmented from 200 to 300 rpm due to the breakup of the aggregates at the high AR and their transformation into smaller structures.

The removal efficiencies of NH_3_-N improved when AR augmented from 100 to 300 rpm. At high AR, more air and H_2_ gas bubbles generated by water reduction in the cathode electrode have been released into the solution environment. They caused the ammonia in the solution environment to be removed.

Although EC process has proved to be effective in treating landfill leachate, there are certain limits in its application to the Bingöl landfill leachate, which are identical to the issues faced in treating other leachates with EC. These limitations are as follows:

1. Considerably, the electrode is the primary affecting factor EC process. It is necessary to regularly replace the ‘sacrificial anode’ throughout the EC process because the mechanism of EC causes anode to continually dissolve in order to produce metal ions, which function as a flocculant.
2. It is also possible to result in secondary contamination due to the release of metal ions into the wastewater.
3. EC failure will occur continuously in the cathode due to the formation of an oxide layer on its surface as the reaction progresses.
4. A significant drawback of EC, aside from electrode effect, is the possibility of sludge formation throughout the process.

Studies have shown that the resulting sludge includes a high concentration of metal ions, including aluminium and iron, along with other recalcitrant contaminants (Guo et al. 2022). For several reasons, it will be challenging to clean the sludge that subsequently develops (Fernandes et al. 2015).

### *The reaction kinetics and reaction rate constants for the COD, NH*_*3*_*-N and turbidity disposal*

**Fig. 9:**
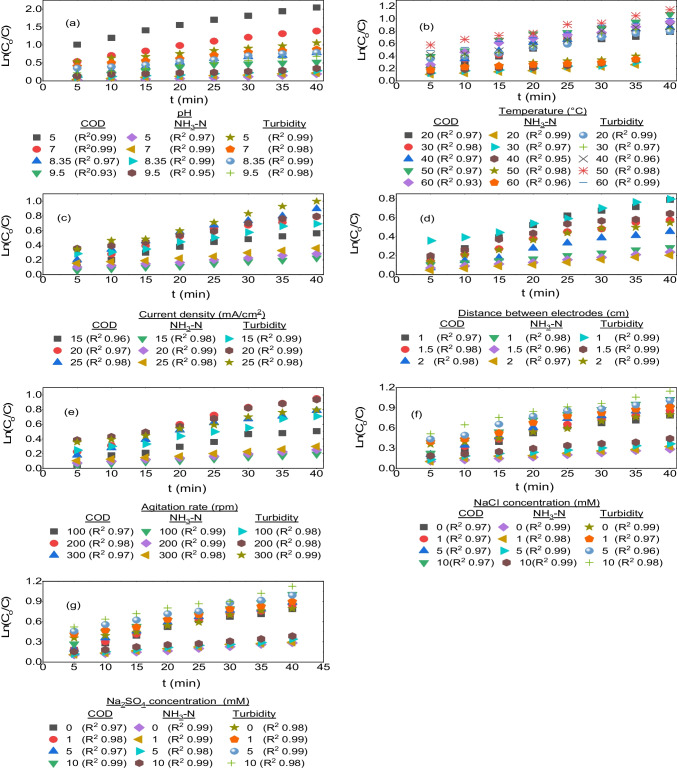
The PFO reaction rate kinetics of COD, NH_3_-N and turbidity parameters for the leachate, depending on initial pH (**a**) and temperature (**b**), CD (**c**), DBE (**d**), AR (**e**), and NaCl (**f**) and Na_2_(SO_4_) (**g**) concentrations

**Fig. 10:**
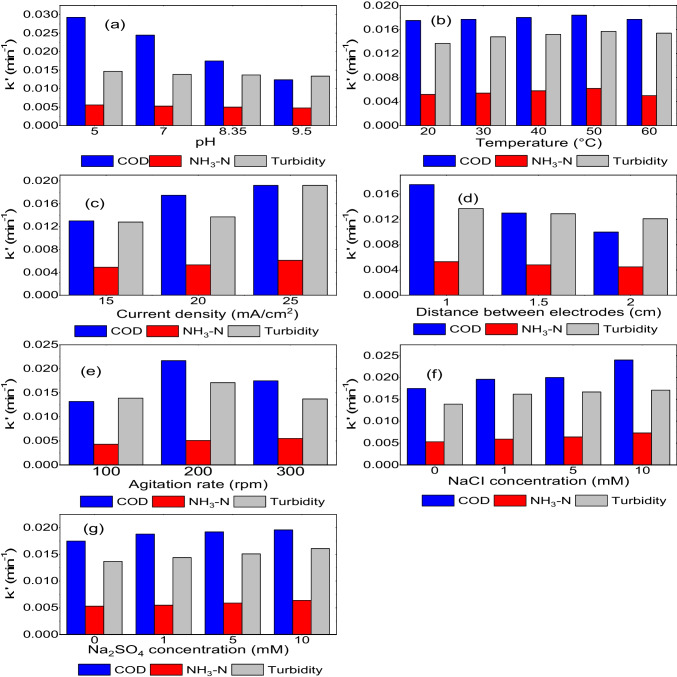
The PFO reaction rate constant values of COD, NH_3_-N and turbidity parameters for the leachate, depending on initial pH (**a**) and temperature (**b**), CD (**c**), DBE (**d**), AR (**e**), and NaCl (**f**) and Na_2_(SO_4_)_2_ (**g**) concentrations

To eliminate the required pollutants at the proper level and determine the hydraulic retention period in the reactor, kinetic studies of the reaction are necessary in every wastewater treatment reactor. As a result, determining the response rate constant is essential for creating wastewater treatment systems. The rate of change in the concentration of the reacting substance per unit of time is known as the reaction rate. High correlation coefficients (*R*^2^ ≥ 0.93–0.99) in the removal of COD, NH_3_-N and turbidity from the leachate as a function of solution pH, temperature, CD, DBE, AR and electrolyte concentrations of NaCl and Na_2_SO_4_ indicated that reaction kinetics matched PFO kinetic model. Equation *V* = *k*′[*C*] represents the PFO reaction kinetics. When the equation is integrated at (*t* = *t*_0_ and *t* = *t*) as well as (*C* = *C*_0_ and *C* = *C*), its linear version ln(*C*_0_/*C*) = *k*′*t* is obtained. *C*_0_ and C, t, and k′ are the COD (mg O_2_/L), NH_3_-N (mg/L), and turbidity (mg (NH_2_)_2_H_2_SO_4_/L) concentration at the beginning and any time, time in min, and PFO reaction rate constant in min^−1^, respectively. Figures 9 and 10 show the reaction kinetics and values of the PFO reaction rate constants obtained under various experimental circumstances.

The rate constant values derived from the COD removal increased with increasing CD, decreasing DBE and pH values and increasing electrolyte concentrations of NaCl and Na_2_SO_4_. Additionally, the reaction rate constant value rose from 20 to 50 °C and reduced from 50 to 60 °C as the temperature of the solution rose.

The PFO reaction rate constant for COD disposal augmented as AR rose from 100 to 200 rpm but reduced as AR rose from 200 to 300 rpm. At a pH value of 5, the biggest reaction rate constant value for COD removal from the leachate was 2.93 × 10^−2^ (min^−1^).

The calculations for NH_3_-N removal revealed that the PFO rate constants increased with pH, temperature, CD, AR, increase in NaCl and Na_2_SO_4_ electrolyte concentration and decrease in electrode distance.

The optimum rate constant value for NH_3_-N removal from leachate was 7.3 × 10^−3^ (min^−1^) for a 10 mM NaCl concentration. As shown in Fig. 10, the PFO rate constants increased in tandem with the values of all experimental parameters.

The results of the turbidity removal calculations reveal that the PFO rate constant values increased with increasing CD, decreasing DBE and pH values and increasing electrolyte concentrations of NaCl and Na_2_SO_4_. The rate constant value increased from 20 to 50 °C, but declined from 50 to 60 °C. Also, the PFO reaction rate constant for turbidity removal rose as AR rose from 100 to 200 rpm, but reduced as AR rose from 200 to 300 rpm. When CD was 25 mA/cm^2^, the greatest reaction rate constant value for turbidity removal from leachate was determined as 1.92 × 10^−2^ min^−1^.

### Calculation of operating cost for the COD disposal from leachate

An important step in the EC process is calculating the operational cost. Energy consumption (kWh/m^3^), unit energy consumption (kWh/kg COD), unit electrode consumption cost ($/kg COD) and total consumption cost ($/kg COD) were calculated within the scope of this study by determining the operating costs for the removal of the COD parameter based on the applied current density and leachate process time. Cost analyses were performed under the following experimental conditions: pH 8.35, 20 °C, 300 rpm, 1 cm, electrolyte-free environment, 40-min reaction period and 400 mL sample volume.

Table 2 shows the electricity consumption, unit energy consumption and total electrode cost estimations based on the applied current densities of the EC processes. Table 2 shows that increasing CD in the EC process from 15 to 25 mA/cm^2^ augmented energy consumption from 80.12 to 218.56 kWh/m^3^, the unit energy consumption from 9.602 to 36.22 kWh/kg COD and total consumption cost from 1.134 to 3.882 $/kg COD, respectively.

**Table 2:** The cost analysis of the EC processes

Current density (mA/cm^2^)	Energy consumption (kWh/m^3^)	Unit energy consumption (kWh/kg COD)	Electrode cost ($/kg COD)	Total consumption cost ($/kg COD)
15	80.12	9.602	0.27	1.134
20	144	19.913	0.415	2.207
25	218.56	36.222	0.622	3.882

## Conclusions

COD and turbidity removal efficiency from the leachate increased with increasing AR, temperature, NaCl, Na_2_SO_4_ and CD, and decreasing pH and DBE. NH_3_-N removal efficiency from the leachate increased with increasing pH, AR, temperature, NaCl, Na_2_SO_4_ and CD, and decreasing DBE. The turbidity and COD disposal efficiency increased as the initial pH value decreased. At pH 5 values, the optimum disposal efficiencies for COD and turbidity from the leachate were determined to be 87% and 62%, respectively. Following a 40-min reaction, the BOD_5_ concentration and BOD_5_/COD ratio were determined to be 85.75 mg O_2_/L and 0.64, respectively, at pH 5. At a NaCl concentration of 10 mM, the optimum disposal efficiency for NH_3_-N from the leachate was determined to be 33%. At temperatures ranging from 20 to 50 °C, removing turbidity, NH_3_-N and COD from the leachate was successful. However, when the initial temperature increased from 50 to 60 °C, the removal efficiency of COD, NH_3_-N and turbidity decreased due to floc disintegration. As the AR increased from 100 to 200 rpm, the COD and turbidity disposal increased. However, when the AR increased from 200 to 300 rpm, COD and turbidity disposal decreased. NH_3_-N removal, on the other hand, increased when the AR increased from 100 to 300 rpm.

Due to high correlation coefficients (*R*^2^ = 0.93–0.99) in removing COD, NH_3_-N and turbidity from the leachate as a function of pH, temperature, CD, DBE, AR, and NaCl and Na_2_SO_4_ concentrations, reaction kinetics matched the PFO kinetics. The highest PFO rate constant values for COD, NH_3_-N and turbidity removal from the leachate were 2.9 × 10^−2^ min^−1^, 1.92 × 10^−2^ min^−1^ and 7.3 × 10^−3^ min^−1^, respectively, at pH 5, NaCl concentration of 10 mM and CD of 25 mA/cm^2^. In the EC process, increasing the CD from 15 to 25 mA/cm^2^ increased energy consumption from 80.12 to 218.56 kWh/m^3^, unit energy consumption from 9.602 to 36.22 kWh/kg COD and total consumption cost from 1.134 to 3.882 $/kg COD.

Findings from studies conducted under different experimental conditions revealed that COD and turbidity removal efficiencies from the leachate will be higher at low pH and high current densities. On the other hand, it was determined that the removal efficiency of NH_3_-N by EC was at low levels. In this research, it was concluded that the EC process applied to remove COD, NH_3_-N and turbidity from the leachate may be used in treatment plants to remove these and similar pollutants under environmental conditions after being scaled up to larger volumes.

## Abbreviations

Al: Aluminium
AMP: Anode material price
AR: Agitation rate
BOD_5_: Biochemical oxygen demand
CD: Current density
*C*_electrode_: Quantity of aluminium
*C*_energy_: Energy consumption
COD: Chemical oxygen demand
DBE: Distance between electrodes
DC: Direct current
EC: Electrocoagulation
EEC: Electrical energy consumption
EEP: Electrical energy price
EOC: Electrical operating cost
*F*: Faraday constant
*I*: Current intensity
*k*′: Pseudo-first-order rate constant
*m*: Amount of aluminium and hydroxide
MC: Cost of electrode material
*M*_w_: Mass weight of the anode
NH_3_-N: Ammonia nitrogen
NTU: Nephelometric turbidity unit
PFO: Pseudo-first-order
PSO: Pseudo-second-order
*t*: Time
TDS: Total dissolved solids
*U*: Voltage
UED: Unit energy demand
*V*: Leachate volume
*Y*_t_: Removal efficiency
*z*: Number of electrons
